# Commentary on a trial comparing krill oil versus fish oil

**DOI:** 10.1186/1476-511X-13-2

**Published:** 2014-01-02

**Authors:** Peter D Nichols, Soressa M Kitessa, Mahinda Abeywardena

**Affiliations:** 1 CSIRO Food Futures Flagship, Marine and Atmospheric Research, Hobart, TAS, Australia; 2 CSIRO Animal, Foods and Health Sciences, Adelaide, South Australia, Australia; 3 CSIRO Preventative Health Flagship Adelaide, Adelaide, South Australia, Australia

**Keywords:** Krill oil, Fish oil, Fatty acids, Trial

## Abstract

Considerable interest exists presently in comparing the performance of krill oil (KO) and fish oil (FO) supplements. Ramprasath et al. (*Lipids Health Dis***12:**178, 2013) have recently compared use of KO and FO in a trial with healthy individuals to examine which oil is more effective in increasing n-3 PUFA, decreasing the n-6:n-3 ratio and improving the omega-3 index. The authors concluded that KO was more effective than FO for all three criteria. However, careful examination of the fatty acid profiles of the oils used showed that the FO used was not a typical FO; it contained linoleic acid as the dominant fatty acid (32%) and an n-6:n-3 ratio of >1. Due to the fatty acid profile being non-representative of typically commercially marketed FO, the conclusions presented by Ramrasath et al. (*Lipids Health Dis***12:**178, 2013) are not justified and misleading. Considerable care is needed in ensuring that such comparative trials do not use inappropriate ingredients.

## Background

The benefits of the long-chain (≥C_20_) n-3 oils (LC n-3 oils) for reduction of the risk of a range of disorders including coronary heart disease, stroke and arthritis is recognised and well documented [1-7]. It is clear that the benefits result from eicosapentaenoic acid (EPA, 20:5n-3) and docosahexaenoic acid (DHA, 22:6n-3), and optimal intake levels of these bioactive fatty acids for maintenance of health and for prevention and treatment of specific diseases have been developed and adopted by both national and global health agencies. These developments have lead to a steady increase in consumer demand for the LC n-3 oils, mainly in the form of fish oil supplements. The increasing global population has substantial implication for the future sustainability of wild harvest fish stocks to meet this demand. Alternate sources of the LC n-3 oils are being explored and developed. KO is one such oil that has captured increasing consumer interest and market share. Compared to FO which contains predominantly triacylglycerol (TAG), KO contains EPA and DHA in both TAG and phospholipid (PL) form [8]. Interest has existed on whether the phospholipid form of the LC n-3 oils is more bioavailable than the TAG form.

Hence, a recent study by Ramprasath et al. [1] aimed to compare the relative effects of KO versus FO against a placebo (corn oil) on plasma and RBC fatty acid profiles in healthy volunteers following 4 wk of supplementation.

## Fish oil – fatty acid profile

Fish oils are the major recognized sources of LC n-3 oils, predominately EPA and DHA, with n-6 fatty acids such as linoleic acid (LA, 18:2n-6) and arachidonic acid (ARA, 20:4n-6) typically only minor components. FO generally show an n-6/n-3 ratio of <1, usually <0.2 (Table 1). The 18/12 FO preparations commercially available are reflective of this since the n-6 fatty acid levels range from 2.9-3.6%.

**Table 1:** **Major fatty acid composition of krill and fish oil used in Ramprasath et al.**[1]**and in typical fish oil**

	**Ramprasath et al. [**[1]**]**		**Typical profile for fish oil**
**Fatty acid**	**Krill oil**	**Fish oil**	
16:0	22.1	17.1	15.4
18:2n-6 LA	2.1	32.5	1.4
18:1n-9	13.3	2.6	8.8
18:0	1.4	3.5	3.4
20:5n-3 EPA	16.4	13.5	19.1
22:6n-3 DHA	9.5	8.7	11.5
n-6:n-3 Ratio	0.095	1.2	0.14
EPA + DHA consumed*	778	664	918

*Calculated from oil compositions and daily consumption of 3000 mg of oil.

Typical fish oil data is the mean of 4 representative 18/12 product brands analysed following transmethylation and GC [9].

In contrast, the profile of the FO used by Ramprasath et al. [1] (Table 1) shows linoleic acid (LA, 18:2n-6, 32%) to be clearly the dominant fatty acid followed by 16:0 (17%), EPA (13.5%) and DHA (8.7%). The source of the oil was stated as a TG 18/12 oil. The n-6/n-3 ratio was 1.2. The typical 18/12 TG oils generally contain 18% EPA and 12% DHA, with LA at <2% (Table 1).

It is well known that n-3 and n-6 essential fatty acid series compete with each other for further metabolism. The use of the FO with a high LA level as described by Ramprasath et al. [1] has resulted in lower LC n-3 and a markedly increased n-6/n-3 ratio than would be expected with a ‘standard or typical’ FO preparation which generally contain only <2% LA. The authors reported that the use of KO with healthy individuals was more effective in increasing n-3 PUFA, decreasing the n-6:n-3 ratio and improving the omega-3 index. Calculation of the amount of EPA + DHA consumed by the two groups of volunteers in the study by Ramprasath et al. [1] shows that the KO group received 114 mg/day higher amounts of the two n-3 LC-PUFA (778 mg v 664 mg, Table 1) without taking into account any competitive actions imposed by the presence of high level of LA (32%) in the FO treated group. Collectively, these major differences are likely to be responsible for the greater incorporation of n-3 PUFA following consumption of KO compared to the FO group. Unfortunately the trial has been biased by use of an oil which was appears to be a mixture of a FO product (Table 1) diluted or blended with an oil enriched in LA.

Accordingly the trial, which was designed to compare the bio-efficacy of incorporation of n-3 PUFA derived from KO and FO, would need to be repeated using a fully verified standard FO product that conforms to specifications presented above. In a more general context, considerable care is required with both product verification and subsequent trial design to ensure that stated aims can be realistically tested and achieved.

## Abbreviations

ARA: Arachidonic acid; DHA: Docosahexaenoic acid; EPA: Eicosapentaenoic acid; FO: Fish oil; GC: Gas chromatography; KO: Krill oil; LA: Linoleic acid; LC: Long-chain (≥C_20_); LC-PUFA: Long-chain polyunsaturated fatty acids; PUFA: Polyunsaturated fatty acids; RBC: Red blood cells

## Competing interests

PDN, SMK and MA declare no conflict of interest.

## Authors’ contributions

PDN analyzed and collated the typical FO data. All authors shared analysis of data and manuscript preparation. All authors read and approved the final manuscript.
